# A Low-Noise, Low-Power, and Wide-Bandwidth Regulated Cascode Transimpedance Amplifier with Cascode-Feedback in 40 nm CMOS †

**DOI:** 10.3390/s26020465

**Published:** 2026-01-10

**Authors:** Xiangyi Zhang, Yuansheng Zhao, Guoyi Yu, Zhenghao Lu, Chao Wang

**Affiliations:** 1 Wuhan National Laboratory for Optoelectronics, Huazhong University of Science and Technology, Wuhan 430074, China; 2 School of Integrated Circuits, Huazhong University of Science and Technology, Wuhan 430074, China; 3 School of Optical and Electronic Information, Huazhong University of Science and Technology, Wuhan 430074, China; 4 School of Electronic Information, Soochow University, Suzhou 215031, China

**Keywords:** transimpedance amplifier, regulated cascode, cascode-feedback, capacitive degeneration, bandwidth extension

## Abstract

The dramatic growth in the emerging optical applications, including Lidar, short-range optical communication, and optical integrated sensing and communication (ISAC) calls for high-bandwidth transimpedance amplifiers (TIA) with low noise and low power in advanced CMOS technology nodes. To address the issues of existing TIA design, including the conventional RGC structure and the dual-feedback regulated cascode (RGC) TIA, design with complex feedback paths, i.e., limited bandwidth, extra noise, and high power consumption for enough bandwidth, this paper presents a novel TIA with the following key contributions. A novel RGC structure with cascode-feedback is proposed to increase feedback gain, thereby extending bandwidth and reducing noise. Design strategy of the proposed RGC TIA in a low-power advanced CMOS process is carried out to exploit weak inversion operation to achieve better power efficiency. Frequency response and noise analysis are also conducted to achieve target bandwidth and noise performance. The proposed TIA is designed and simulated in 40 nm CMOS with a target PD capacitance of 0.15 pF, achieving a −3 dB bandwidth of 9.2 GHz and a transimpedance gain of 71 dBΩ. The average input-referred noise current spectral density is 18.3 pA/$\sqrt{Hz}$. Operating at 1.2 V, the core circuits consume only 6.6 mW, excluding the output buffer. Compared with prior RGC TIA designs, the proposed TIA achieves a 7.4×~243× enhancement in figure of merit.

## 1. Introduction

**Figure 1:**
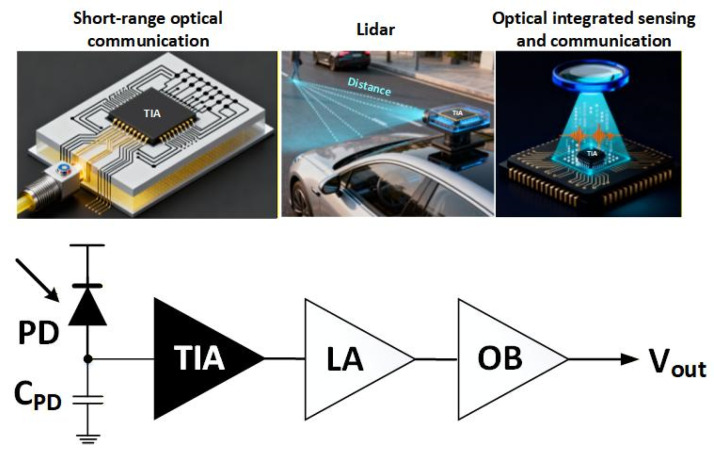
Applications of TIAs in the front end of an optical receiver.

With the dramatic increase in the emerging optical applications, including Lidar, short-range optical communication, optical wireless communication, as well as optical integrated sensing and communication (ISAC) [1,2,3,4,5,6], the demand for high-speed optical receivers is growing rapidly. In the optical receivers, the analog front-end (AFE) determines the key performance of the whole transmission system, such as bandwidth and noise [7,8]. Figure 1 shows the front-end of an optical receiver, consisting of a photodiode (PD), a transimpedance amplifier (TIA), a limiting amplifier (LA), and an output buffer (OB) [9]. As the key optoelectronic interface, the PD transforms the received optical signal into a weak photo-current signal. The TIA then converts the weak photo-current signal to a normal voltage signal with a good signal-to-noise ratio (SNR), which is further amplified by the LA and driven by OB for post-processing. As the first stage of the receiver AFE circuit, the TIA faces a formidable challenge in achieving high bandwidth for high-speed data transmission while ensuring low input-referred noise current for sensitivity and low power consumption to minimize the operating cost of the optical system [10].

Traditionally, the AFE circuits and devices are fabricated by conventional III/V or SiGe technologies for their speed and noise advantages. However, those technologies are no longer suitable for high-integration-density and cost-sensitive applications. Advanced CMOS process has become more popular for its lower cost and higher integration density; however, the lower supply voltage poses a significant challenge to broadband circuit design.

In TIA designs, bandwidth is mainly restricted by the large PD parasitic capacitance $C_{PD}$ at the input node [11,12], especially the inverter-based TIA with shunt-shunt feedback in [13,14,15,16]. To realize high-bandwidth TIAs, different topologies for reducing input impedance $\left(R_{in}\right)$ have been adopted. The regulated cascode (RGC) TIA is designed in [17,18] to reduce $R_{in}$ and thus the bandwidth-limiting impact of $C_{PD}$. The dual-feedback modified RGC TIA in [19,20,21] further reduces $R_{in}$ by an additional feedback path. Nevertheless, this comes at the cost of decreased gain due to the output resistance $\left(R_{out}\right)$ reduction. In [22,23], the dual-feedback structure is incorporated with the current-reuse technique to decouple the $R_{in}$-gain trade-off. However, both modified RGC structures with complex feedback paths severely limit the bandwidth and also inevitably introduce notable noise, which is the first design challenge in high-bandwidth RGC TIA designs.

For low-frequency analog circuit design in conventional CMOS technology, transistor channel lengths are normally sized around or above 1 μm to achieve high output resistance $r_{o}$. In contrast, for high-frequency wide-bandwidth circuit design in advanced CMOS technology, transistors are sized to the minimal channel length to ensure adequate cut-off frequency $f_{T}$, whereas $r_{o}$ is significantly degraded. In addition, the RGC topology in [18,23,24] requires large voltage headroom to bias the transistors in strong inversion to achieve high $f_{T}$, which is not feasible in advanced CMOS processes with low supply voltage near 1 V, limiting the bandwidth performance through three aspects of feedback gain, input resistance, and the Miller effect. Furthermore, the modified RGC TIA designs operating at low voltage in [21,25,26] require large bias current to ensure high $f_{T}$ of the transistors in strong inversion, increasing the overall power consumption. Therefore, the second design challenge lies in the constraint faced by low-power RGC TIAs when implemented in advanced CMOS technology with low supply voltages.

To solve the aforementioned major design challenges, this paper proposes a novel RGC TIA to overcome the bandwidth limitations faced by the existing RGC TIA and at the same time achieve low power and low noise in an advanced CMOS process (the basic TIA circuit was first briefly presented at the ICCS2023 conference [27]). The major contributions of this work are listed as follows:

(1) To address the issues of limited bandwidth and noise in the existing designs such as the dual-feedback RGC TIAs, a single-stage cascode feedback structure is employed as the feedback amplifier in the proposed RGC input stage to boost feedback gain and also reduce Miller capacitance at the input node. By increasing the pole frequency, the bandwidth is dramatically extended for isolating the large $C_{PD}$ from bandwidth determination. To balance the bandwidth, gain, and noise, a bandwidth-extension stage with capacitive degeneration and three gain-boosting stages with near-zero noise are cascaded after the RGC input stage with negligible noise contribution to the proposed TIA design.
(2) To address the issues of high supply voltage and bias current in the existing RGC TIA works with transistors working in strong inversion for sufficient $f_{T}$, the design strategy under low supply voltage near 1 V required by advanced process technology below 65 nm is discussed. The design strategy suggests biasing the common-source (CS) feedback transistor in weak inversion to achieve sufficient voltage headroom and improve the power and noise efficiency of the CS transistor with enough $f_{T}$ in advanced CMOS technology. Therefore, the design strategy enables the proposed TIA to achieve considerable bandwidth and much lower power under low supply voltage.
(3) To characterize the bandwidth and noise of the proposed RGC TIA, the frequency response and noise analysis are carried out. By the pole-zero analysis based on derived pole-zero equations and precise setting of the pole-zero frequencies, the proposed TIA achieves a flat frequency response within a wide bandwidth. In addition, by the noise analysis based on the derived input-referred noise current of the RGC TIA, the noise-critical devices are identified, and therefore, the dimensions of these key devices are optimized for effective noise reduction.

The paper is organized as follows: In Section 2, the proposed RGC TIA is presented. Section 2.1 and Section 2.2 discuss the modified RGC input stage with the design strategy under low supply voltage, corresponding to contributions (1) and (2), respectively. Section 2.3 introduces the following bandwidth extension and gain-boosting stages. Section 2.4 and Section 2.5 elaborate on the frequency response and noise analysis for contribution (3). Section 3 presents the simulation results and the comparison with the existing 10 Gb/s TIA designs in CMOS technology. Finally, Section 4 draws the conclusions.

## 2. Modified RGC TIA Design

**Figure 2:**
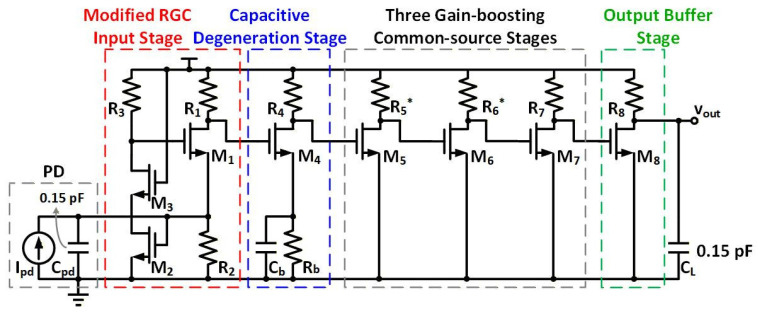
Schematic of the proposed cascode-feedback RGC TIA. * Trimmed resistors detailed in Section 3.

Figure 2 depicts the schematic of the proposed broadband RGC TIA, consisting of a single-stage cascode-feedback RGC input stage ($M_{1}\sim M_{3}$), a bandwidth-extension stage with capacitive degeneration ($M_{4}$), three gain-boosting CS stages ($M_{5}\sim M_{7}$), and a CS output buffer ($M_{8}$) for impedance matching and output measurement. At the input node, a current source $I_{\mathrm{pd}}$ and a capacitor $C_{\mathrm{pd}}$ of 0.15 pF [28] are connected in parallel to simulate the output current and junction capacitance of the PD, respectively. A load capacitance $C_{L}$ of 0.15 pF is connected between the TIA output and ground for simulating the pad capacitance and the loading capacitance of subsequent circuits.

### 2.1. Cascode-Feedback RGC Input Stage

In this section, the single-stage cascode feedback mentioned in contribution 1 is discussed in detail.

**Figure 3:**
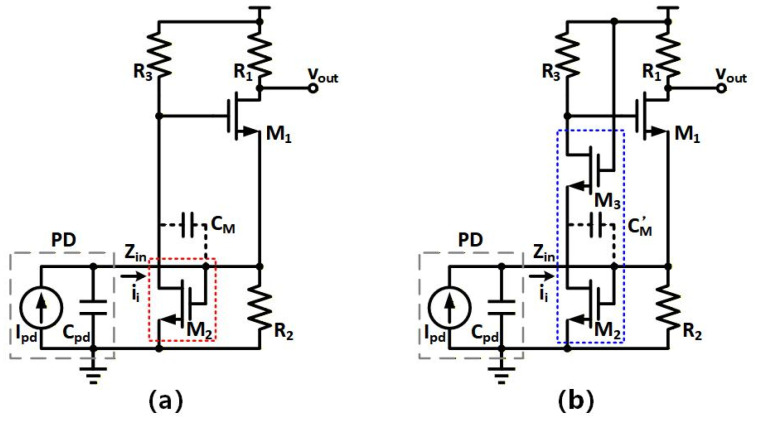
(**a**) conventional RGC structure; (**b**) proposed single-stage cascode-feedback RGC structure.

Figure 3a shows the conventional RGC structure consisting of the common-gate input transistor $\left(M_{1}\right)$ and the CS feedback amplifier (M_2_ & R_3_), which provides a low input impedance with strong local feedback. The transimpedance gain $R_{T}$ and the input resistance $R_{\mathrm{in}}$ are shown as [29]

(1) $$R_{T}=Z_{T,\mathrm{RGC}}\left(0\right)\;\text{≈}\;R_{1},$$

(2) $$R_{\mathrm{in}}=Z_{\mathrm{in},\mathrm{RGC}}\left(0\right)\;\text{≈}\;\frac{1}{g_{m1}\left(1+A\right)},$$

in which A is the gain of the CS amplifier and g_m1_ is the transconductor of M_1_.

The pole frequency located at the input node is determined by the input impedance R_in_ and the lumped capacitance, which is given by

(3) $$\omega_{in}=\frac{1}{R_{in}[C_{PD}+C_{M}+C_{g2}+C_{s1}]},$$

where $C_{PD}$, $C_{M}$, $C_{g2}$ and $C_{s1}$ is the PD capacitance, the Miller capacitance resulted from $C_{gd2}$, $C_{gb2}+C_{gs2}$, and $C_{gs1}+C_{ds1}$, respectively. A lower input impedance can help to increase the input node’s pole frequency even with a large $C_{PD}$.

Based on the aforementioned principle analysis of conventional RGC in Figure 3a, the proposed RGC in Figure 3b is specifically designed to utilize the single-stage cascode feedback (M_2_ & M_3_) to provide a higher feedback gain for reducing both input resistance and the Miller effect under low supply voltage through three aspects as below.

Firstly, the gain of the proposed RGC input stage is boosted by the cascode feedback. As depicted in Figure 3a, the attainable gain A of CS feedback amplifier (M_2_ & R_3_) in the conventional RGC is shown as

(4a) $$Z_{m}=\frac{V_{g1}}{I_{in}}=\frac{-(+R_{2}I_{pd}g_{m2})(R_{3}//r_{o2})}{I_{pd}}=-R_{2}g_{m2}(R_{3}//r_{o2})<0,$$

(4b) $$A=\frac{V_{g1}}{V_{in}}=\frac{-(+R_{2}I_{pd}g_{m2})(R_{3}//r_{o2})}{R_{2}I_{pd}}=-g_{m2}(R_{3}//r_{o2})\approx -g_{m2}R_{3}/2<0,$$

where $V_{g1}$ is the node voltage at the gate of $M_{1}$ and $r_{o2}$ is the output resistance of $M_{2}$. When M_2_ is sized to around minimal channel length to reduce parasitics and power, $r_{o2}$ becomes as large as $R_{3}$, thereby halving the gain as compared to the long channel length case. In order to compensate the gain reduction, the cascaded transistor $M_{3}$ in Figure 3b is employed to increase A to A′, as described by

(5) $$A^{\prime }=-g_{m2}(R_{3}//\{r_{o3}+r_{o2}[1+(g_{m3}+g_{mb3})r_{o3}]\}\approx -g_{m2}(R_{3}//g_{m3}r_{o3}r_{o2})\approx \;-g_{m2}R_{3}=2A<0,$$

Secondly, the input resistance of the proposed RGC input stage is reduced by the cascode feedback. As shown in Figure 3a, the low supply voltage around 1 V forces the CS feedback transistor M_2_ to be sized wider to have a larger bias current for achieving sufficient transconductance $g_{m}$, leading to a significantly higher power and a larger parasitic capacitance. For low-voltage operation at 1.2 V, the CS transistor $M_{2}$ of the proposed cascode feedback in Figure 3b is biased in the weak inversion region instead so as to achieve sufficient voltage headroom and also exploit a higher transconductance efficiency of $g_{m2}/i_{2}$ in the sub-threshold operation of M_2_. Therefore, $M_{2}$ can achieve a large $g_{m2}$ with a small bias current $i_{2}$ under a low voltage of 1.2 V, thereby having a large feedback gain to reduce the input resistance R_in_.

Thirdly, the Miller effect of the proposed RGC input stage is reduced by the cascode feedback. As shown in Figure 3a, a wide channel width of M_2_ is to achieve a sufficient g_m2_ and feedback gain, but it introduces a large Miller capacitance at the input node of the conventional RGC, as described by

(6) $$C_{M}=(1+A)C_{gd2},$$

where C_M_ is the Miller capacitance and C_gd2_ is the gate-drain capacitance of M_2_. In the proposed cascode feedback in Figure 3b, the Miller effect of $M_{2}$ in weak inversion is reduced by the cascaded transistor $M_{3}$, as described by

(7) $$C_{M}^{\prime }=(1+g_{m2}/g_{m3})C_{gd2},$$

where $g_{m2}$ is comparable to $g_{m3}$. Therefore, the Miller capacitance is around two times larger than C_gd2_, which is significantly smaller than that without $M_{3}$, as described by Equation (7).

Based on the above analysis, Table 1 compares the key factors limiting bandwidth of the proposed RGC input stage in Figure 3b and the conventional RGC structure in Figure 3a. By reducing input resistance $R_{in}$ and Miller effect $C_{M}$ under low supply voltage of 1.2 V, the proposed design pushes the input node pole frequency to a higher frequency range, thus achieving a broader bandwidth without introducing a large amount of extra noise or power overhead.

### 2.2. Design Strategy Under Low Supply Voltage

In the RGC input stage, the gain of feedback loop can be derived as

(8) $$A=g_{m2}R_{3}=g_{m2}/i_{2}\times i_{2}R_{3},$$

which requires a large transconductance efficiency $g_{m2}/i_{2}$ of $M_{2}$ and a large voltage drop on $R_{3}$. The supply voltage of the proposed RGC structure is shown as

(9) $$Supply\;voltage=V_{DD}=i_{2}R_{3}+V_{gs1}+V_{gs2},$$

where $V_{gs2}$ equals to $i_{1}R_{2}$, the voltage drop on $R_{2}$.

The output voltage and swing of the RGC stage are shown as

(10a) $$V_{out}=V_{DD}-i_{1}R_{1},$$

(10b) $$Output\;swing=2i_{1}R_{1},$$

where $i_{1}R_{1}$ is the voltage drop on $R_{1}$. Constrained by the saturation-region operation of $M_{1}$, the output swing can be recalculated by

(11a) $$V_{out,max}=V_{DD},$$

(11b) $$V_{out,min}=V_{g1}-V_{TH1}=V_{DD}-i_{2}R_{3}-V_{TH1},$$

(11c) $$Output\;swing=V_{out,max}-V_{out,min}=i_{2}R_{3}+V_{TH1},$$

where $V_{g1}$ and $V_{TH1}$ are the gate voltage and threshold voltage of $M_{1}$, respectively. By equating (10b) and (11c), the voltage drop on $R_{1}$ is described by

(12) $$2i_{1}R_{1}=Output\;swing=i_{2}R_{3}+V_{TH1},$$

According to Equations (8) and (9), with the $g_{m}/i_{d}$ plus the low supply voltage requirements, $M_{1}$ is biased in strong inversion to achieve high $f_{T}$, while $M_{2}$ is biased in weak inversion for better power and noise efficiency, and their $g_{m}/i_{d}$ values can be set accordingly. $V_{gs}$ can be calculated from $g_{m}/i_{d}$ method. Therefore, $R_{3}$’s voltage drop $i_{2}R_{3}$ is determined by Equation (9) and then A is obtained by Equation (8). Having known A, $g_{m1}$ and $i_{1}$ can be set by choosing the wanted value 50 Ω of input impedance in Equation (2), then the channel width $w_{1}$ of $M_{1}$ device can be obtained. Since $i_{2}R_{3}$ is constant, a larger $R_{3}$ with a smaller $i_{2}$ consumes less power. Similarly, with certain $g_{m2}$ and $i_{2}$, the width $w_{2}$ of $M_{2}$ device can also be derived.

In contrast to the conventional RGC structure with both $M_{1}$ and $M_{2}$ working in strong inversion, the proposed RGC structure biases $M_{2}$ in weak inversion, which contributes a lower $V_{gs2}$ and thus enables a larger $i_{2}R_{3}$, as shown in (9). Therefore, a higher feedback gain A is achieved via (8), and a larger output swing is obtained from (12). In (12), since $i_{1}$ is pre-determined by $g_{m1}/i_{1}$ (for $M_{1}$’s saturation-region operation) and $g_{m1}$ (for 50 Ω input impedance), the larger output swing $2i_{1}R_{1}$ enables a larger $R_{1}$, thereby achieving a higher transimpedance gain of the RGC structure, as depicted in (1).

### 2.3. Bandwidth Extension and Gain-Boosting Stage

Limited by the large $C_{PD}$, the bandwidth of the single-stage cascode-feedback RGC input stage can hardly be further extended without the degradation of gain and noise performance. Therefore, the capacitive degeneration technique is utilized in the bandwidth extension stage to compensate the dominant pole $\omega_{d}$ with a zero $\omega_{z}$ at ${{(R}_{b}C_{b})}^{-1}$. The detailed pole-zero analysis is presented in the next section, Section 2.3. Therefore, as the dominant pole is compensated by the zero, the −3 dB bandwidth of the TIA is determined by the second lowest pole in the TIA and further extended with minimal noise and area overhead.

Note that $R_{b}$ in the capacitive degeneration trades gain for bandwidth, and therefore, three gain-boosting CS stages are employed to compensate for the gain loss. Moreover, considering the load resistors of the gain-boosting stages degrades the bandwidth performance, $\omega_{z}$ is set to a lower frequency than $\omega_{d}$ to realize pre-equalization in the transimpedance response of the proposed TIA.

### 2.4. Frequency Response

The dc transimpedance gain of the proposed TIA is given by

(13) $$Z_{T}(0)\;\approx \;R_{1}\cdot (-\frac{g_{m4}R_{4}}{1+g_{m4}R_{b}})\cdot (-g_{m5}R_{5})\cdot (-g_{m6}R_{6})\cdot (-g_{m7}R_{7})\cdot (-g_{m8}R_{8}),$$

In (13), $-g_{mx}R_{x}$ is the voltage gain of CS stage formed by $M_{x}$ and $R_{x}$, where x is from 5 to 8. $R_{1}$ determines the gain of the cascode-feedback RGC input stage according to (1). In this design, the gain of bandwidth-extension stage ($\frac{g_{m4}R_{4}}{1+g_{m4}R_{b}}$) and the output buffer stage ($g_{m8}R_{8}$) are approximately 1 for pre-equalization and impedance matching, respectively. As a result, the dc gain of the proposed TIA is mainly provided by $R_{1}$ and the CS gain $g_{mx}R_{x}$ of three gain-boosting stages with x from 5 to 7.

In the conventional RGC TIA design, the dominant pole is restricted by large PD parasitic capacitance $C_{PD}$ at the input node. In contrast, as the single-stage cascode feedback further reduces the input impedance $R_{in}$ of the RGC input stage, the dominant pole $\omega_{d}$ of the proposed TIA circuit is located at the drain of $M_{1}$ instead, which is shown as

(14) $$\omega_{d}=\frac{1}{R_{1}(C_{gd1}+C_{db1}+C_{g4})},$$

where $C_{g4}$ is the sum of gate parasitic capacitance of $M_{4}$.

The bandwidth-extension stage with capacitive degeneration is to compensate the dominant pole with a zero at ($R_{b}C_{b}$)^−1^, which requires

(15) $$\omega_{z}=\frac{1}{R_{b}C_{b}}=\omega_{RGC},$$

As $R_{b}C_{b}$ is constant for pole-zero compensation, a relatively large $R_{b}$ with a small $C_{b}$ can be chosen to push $\omega_{b}$ (i.e., $\left((1+g_{m4}R_{b})/R_{b}C_{b}\right)$ generated by the capacitive degeneration) to a higher frequency to prevent $\omega_{b}$ from bandwidth determination.

Therefore, as the dominant pole is compensated in the bandwidth-extension stage, the second lowest pole with the largest capacitance in the gain-boosting and buffer stages determines the −3 dB bandwidth. In the following CS stages, each stage contributes a pole at its output node, as described by

(16) $$\omega_{x}=\frac{1}{R_{x}C_{x}},$$

where $R_{x}$ and $C_{x}$, respectively, refer to the load resistor and the lumped capacitance at the drain of the CS transistor $M_{x}$ with x from 5 to 8.

**Figure 4:**
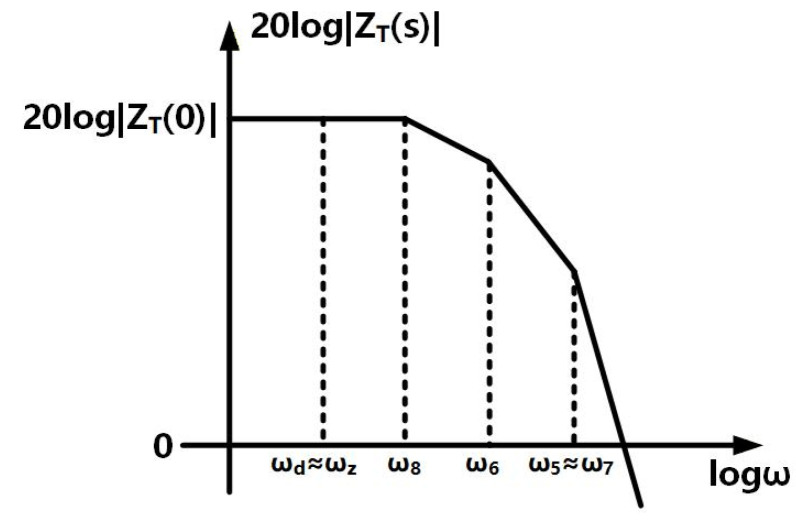
Frequency response of the proposed TIA with the zero and poles.

Based on the aforementioned analysis of zero and poles, the frequency response of proposed TIA design is illustrated in Figure 4. As the frequency increases, the $Z_{T}$ first encounters $\omega_{d}$ and $\omega_{z}$. Since the pole at $\omega_{d}$ is compensated by the zero at $\omega_{z}$, $Z_{T}$ remains constant. Then, when ω exceeds $\omega_{8}$, $Z_{T}$ decreases with a slope of 20 dB/dec. In the three CS stages, as the load resistors satisfy $R_{6}>R_{5}=R_{7}$, thus, $\omega_{6}<\omega_{5}=\omega_{7}$. Therefore, when ω exceeds $\omega_{6}$, $Z_{T}$ further decreases with a slope of 40 dB/dec. When ω exceeds $\omega_{5}$ and $\omega_{7}$, $Z_{T}$ continues to decrease with a slope of 80 dB/dec. Combining Equations (13)–(16), the transfer function of the proposed TIA can be described as

(17) $$Z_{T}\left(s\right)=Z_{T}(0)\frac{1+s/\omega_{z}}{\left(1+s/\omega_{d})(1+s/\omega_{5})(1+s/\omega_{6})(1+s/\omega_{7})(1+s/\omega_{8}\right)},$$

As the lumped capacitance of the NMOS transistors in 40 nm CMOS is less than one-tenth of the load capacitance (150 fF), the second lowest pole of the proposed TIA is located at the output node, which determines the −3 dB bandwidth as described by

(18) $$f_{-3dB}=\frac{\omega_{8}}{2\pi }=\frac{1}{2\pi R_{8}(C_{L}+C_{d8})},$$

where $C_{d8}$ is the drain parasitic capacitance of $M_{8}$, and $C_{L}$ is the output load capacitance.

### 2.5. Noise Analysis

To simplify the analysis, the noise contribution of the stages subsequent to the cascode-feedback RGC input stage is neglected, as their noise is attenuated by the gain of input stage $\left(R_{1}\right)$ when referred to input.

In the cascode-feedback RGC input stage, the noise sources mainly come from the common-gate amplifier ($M_{1}$) and the cascode feedback amplifier ($M_{2}\sim M_{3}$). In general, these device noises are uncorrelated and can be added directly. As a cascaded transistor in the single-stage cascode structure, $M_{3}$ adds no extra noise to the circuit, whose noise contribution is neglected in the following noise analysis. By analyzing the noise sources in the RGC structure, the input-referred noise of the proposed TIA can be expressed as

(19) $$\bar{I_{n,in}^{2}}=\bar{I_{n,R2}^{2}}+\bar{I_{n,M1}^{2}}{\left(s/\omega_{in}\right)}^{2}+\bar{I_{n,R1}^{2}}{\left(1+s/\omega_{in}\right)}^{2}+\frac{1}{g_{m2}^{2}R_{in}^{2}}(\bar{I_{n,R3}^{2}}+\;\bar{I_{n,M2}^{2}}){\left(1+s/\omega_{in}\right)}^{2},$$

where $\bar{I_{n,R2}^{2}}=4KT/R_{2}$, $\bar{I_{n,R1}^{2}}=4KT/R_{1}$, $\bar{I_{n,R3}^{2}}=4KT/R_{3}$, $\bar{I_{n,M1}^{2}}=4KT\gamma g_{m1}$, $\bar{I_{n,M2}^{2}}=4KT\gamma g_{m2}$, K is Boltzmann constant, T is the absolute temperature, γ is the noise factor of MOSFET device. $R_{in}$ and $\omega_{in}$ are derived in Equations (2) and (3), respectively.

From Equation (19), the first term $\bar{I_{n,R2}^{2}}$ is the frequency-independent noise component contributed by $R_{2}$, which is directly added to the input node. The second term, i.e., the noise contribution of $M_{1}$ is extremely small at low frequencies yet increases rapidly with frequency. At high frequencies, the major noise contributors are $M_{1}$, $M_{2}$, and $R_{3}$, with the growth rate primarily determined by the total capacitance at the input node as elaborated in Equation (3). According to (19), the values of $R_{1}$, $R_{2}$, and $R_{3}$ are designed to be as large as possible while still guaranteeing the voltage headroom. And the value of $g_{m1}$ is set considering both noise and bandwidth. Thanks to the proposed design strategy under low supply voltage in Section 2.2, the value of $g_{m2}$ is enabled to be larger at the same current consumption for better noise efficiency.

## 3. Simulation Results and Comparison

Based on the above analysis in Section 2, Table 2 lists the sizing of MOSFET devices in the proposed TIA circuit, which are carefully designed for optimizing the trade-off among bandwidth, gain, and noise performance. The proposed prototype circuit was simulated and analyzed with these dimensions.

**Figure 5:**
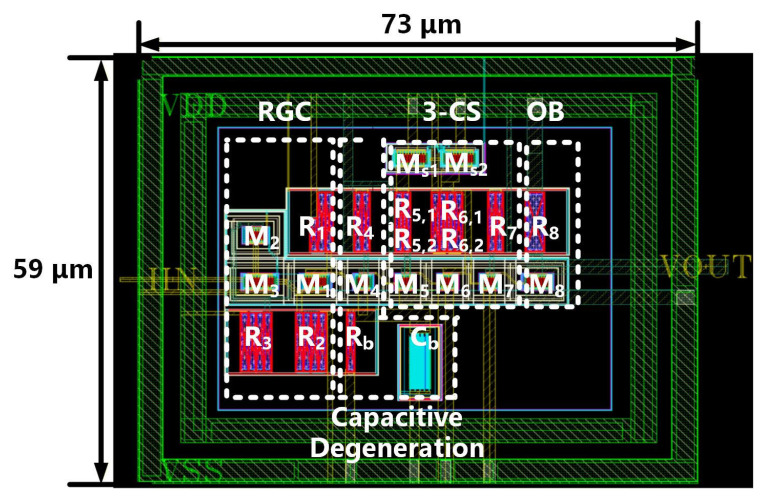
Layout of the proposed TIA circuit in 40 nm CMOS technology.

Figure 5 shows the layout design in 40 nm CMOS technology, and the core area is 0.004 mm^2^. The proposed TIA design is simulated under a 1.2 V supply. The proposed TIA consumes 10.4 mW total power, of which the RGC input stage, capacitive degeneration stage, three CS stages, and output buffer stage account for 1 mW, 1.4 mW, 4.2 mW, and 3.8 mW, respectively. In the first stage, the CS transistor $M_{2}$ is biased in weak inversion for improved power efficiency; therefore, the input stage does not consume high power. The capacitive degeneration stage extends the bandwidth with passive components, i.e., capacitors, thus featuring low power consumption. The three CS stages and output buffer consume more power to boost gain and driving capability, accounting for 40% and 37% of the total energy consumption, respectively. Thanks to the design strategy of the RGC TIA in the 40 nm CMOS process, the proposed TIA consumes 8~72% less power compared with existing 10 GB/s TIA designs in the literature [16,18,23,24,25,26].

**Figure 6:**
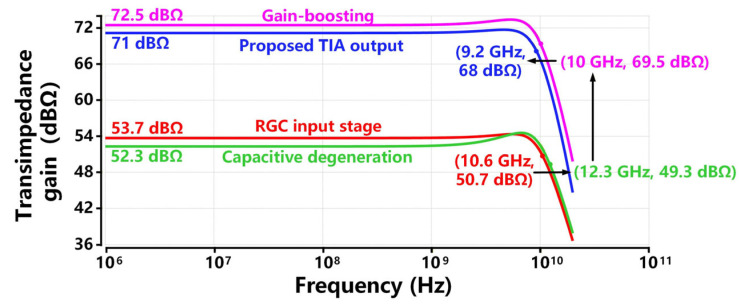
Transimpedance response of each stage in the proposed TIA design.

Figure 6 exhibits the four output node transimpedance responses of the cascode-feedback RGC input stage, capacitive degeneration stage, gain-boosting stage, and output buffer stage from 1 MHz to 20 GHz. As shown in the figure, the proposed TIA achieves a total transimpedance gain of 71 dBΩ from 1 MHz to the high-frequency range. The transimpedance gains simulated at the output nodes of the RGC input stage, capacitive degeneration stage, three CS stages, and output buffer stage are 53.7 dBΩ, 52.3 dBΩ, 72.5 dBΩ, and 71 dBΩ, respectively. Under the constraint of voltage headroom, $R_{1}$ (520 Ω) provides the transimpedance gain of 53.7 dBΩ at the output node of the RGC input stage. Then the capacitive degeneration deteriorates gain by 1.4 dB, trading for a significant bandwidth extension. Therefore, the subsequent three gain-boosting stages increase gain from 52.3 dBΩ to 72.5 dBΩ via the gain product of $\left(g_{m5}R_{5})(g_{m6}R_{6})(g_{m7}R_{7}\right)$. Lastly, the output buffer stage is designed to achieve 50 Ω impedance matching, which results in a 1.5 dBΩ reduction in the overall gain. The result is consistent with the analysis of Equation (13). The proposed TIA achieves an 18 dB~30 dB improvement compared with the existing RGC TIA designs in [18,23,24,25,26], thanks to the help of $R_{1}$ and three CS stages in our design.

Figure 6 shows that the proposed TIA achieves a −3 dB bandwidth of 9.2 GHz with 0.15 pF PD capacitance and 0.15 pF load capacitance $C_{L}$. The simulated −3 dB bandwidths at the output nodes of the RGC input stage, capacitive degeneration stage, three CS stages, and output buffer stages are 10.6 GHz, 12.3 GHz, 10 GHz, and 9.2 GHz, respectively. As the single-stage cascode feedback effectively reduces the input impedance $R_{in}$, the dominant pole is no longer restricted by the large $C_{PD}$ at the input node. In contrast, the bandwidth of the RGC input stage is determined by $R_{1}$ and the parasitic capacitance at the output node. The capacitive degeneration introduces a zero for pre-equalization, further extending the bandwidth from 10.6 GHz to 12.3 GHz, i.e., an enhancement ratio of 1.16. Then, the three CS stages degrade the bandwidth with three poles located at their output nodes, and the output buffer also reduces the bandwidth with the second lowest pole induced by $C_{L}$. The result is consistent with the analysis of frequency response in Section 2.4. Owing to the single-stage cascode feedback and capacitive degeneration techniques, the proposed TIA outperforms the inverter-based TIAs in [16] and RGC TIAs in [18,23] in terms of bandwidth, by 1.8× and 1.15×~1.3×, respectively.

**Figure 7:**
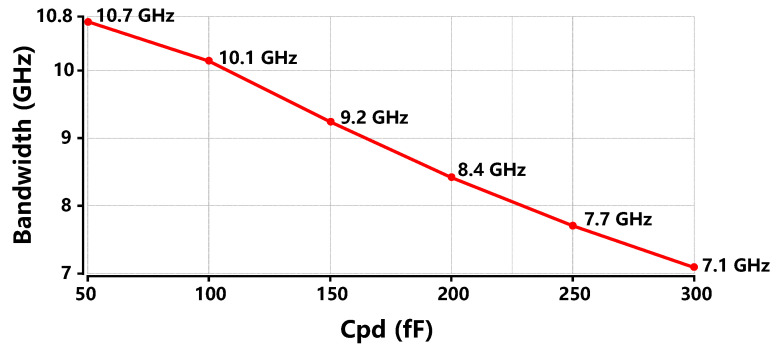
Bandwidth of the proposed TIA with different Cpd values at the input node.

Figure 7 exhibits the bandwidths of the proposed TIA with different $C_{PD}$ values at the input node. As $C_{PD}$ triples from 50 fF to 150 fF, the bandwidth of the proposed TIA decreases from 10.7 GHz to 9.2 GHz, only a reduction of 14%. As $C_{PD}$ doubles from 150 fF to 300 fF, the bandwidth of the proposed TIA decreases from 9.2 GHz to 7.1 GHz, representing a 23% reduction. In contrast, for conventional TIA circuits, the bandwidth would decrease by 50% when $C_{PD}$ doubles. Thanks to the single-stage cascode feedback structure, the impact of the input node on bandwidth is effectively mitigated.

**Figure 8:**
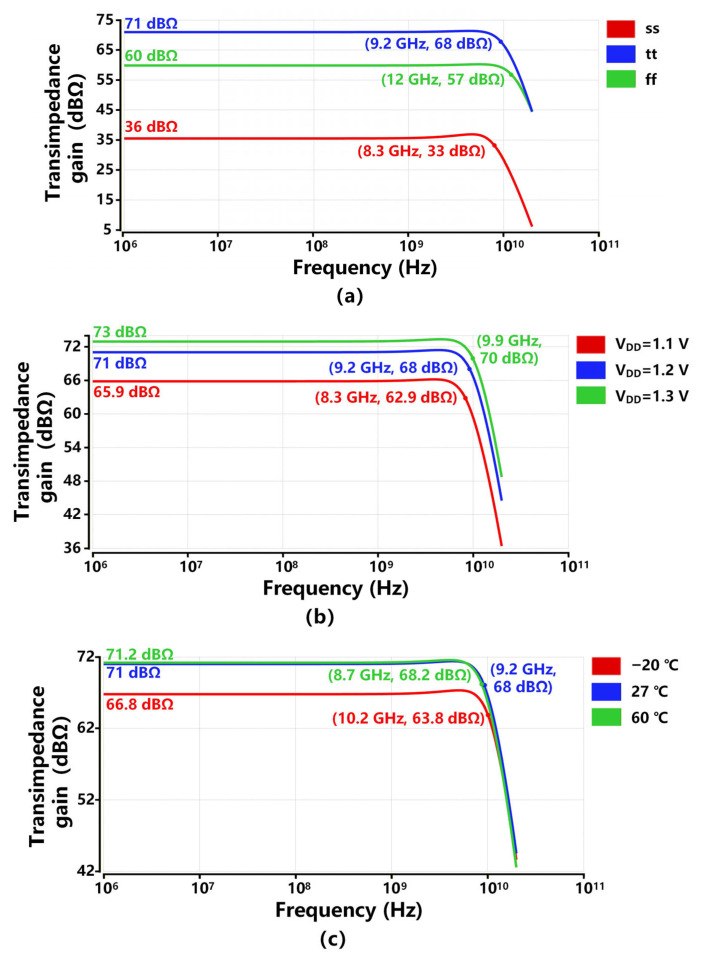
Post-simulated PVT analysis for the transimpedance response of the proposed TIA: (**a**) process, (**b**) supply voltage, (**c**) temperature variations.

To evaluate the process, voltage, and temperature (PVT) variation effects on the proposed TIA, Figure 8 exhibits the transimpedance response of the proposed TIA under three process corners, ±0.1 V of supply voltage variation, and temperature ranging from −20 °C to 60 °C. As illustrated in Figure 8a, the proposed TIA at the ff corner achieves a bandwidth of 12 GHz and a gain of 60 dBΩ. This is attributed to the reduced resistance at the ff corner, thereby increasing the pole frequencies in the TIA circuit. However, the abnormal gain and bandwidth performance at the ss corner are mainly due to increased resistances, which raise the gain of the front-end stages of the proposed TIA, thereby driving the final-stage OB into cut-off operation. This performance degradation caused by process variations can be optimized via the trimming technique, which will be added in our future tape-out. Figure 8b exhibits, for ±0.1 V of supply voltage variation, the transimpedance gain and −3 dB bandwidth changes for 7.1 dBΩ and 1.6 GHz, respectively. The increase of supply voltage results in a higher transimpedance gain and an extended bandwidth. Higher supply voltage produces more current passing through each of the five stages of the proposed TIA for the same $g_{m}/i_{d}$, which enhances $g_{m}$ and thereby boosts the gain and bandwidth as analyzed in Section 2. Figure 8c depicts that for 80 °C variations over temperature, the transimpedance gain and −3 dB bandwidth changes for 4.4 dBΩ and 1.5 GHz, respectively. The increase in temperature results in higher resistances, thereby enhancing the transimpedance gain while decreasing the bandwidth of the proposed TIA. Thanks to the careful design of the sizes and parameters of the critical devices in Section 2, the transimpedance response of the proposed TIA has small variations across different supply voltage and temperature conditions.

Under PVT variations, although the transconductance (g_m_) variations of the common-gate input transistor g_m1_ and the common-source feedback transistor g_m2_ in the RGC input stage are a little bit large, the gain of the RGC input stage is primarily determined by $R_{1}$ in (1), whose variation is not significant. According to (2) and (5), the g_m_ values of the transistors primarily affect the input resistance $R_{in}$ of the RGC input stage, thereby influencing the bandwidth, as shown in (3). Across three process corners, with the supply voltage ranging from 1.1 V to 1.3 V and the temperature spanning −20 °C to 60 °C, $R_{in}$ remains below 100 Ω and S11 is below −10 dB. Therefore, gm variations do not result in significant degradation of gain or bandwidth.

**Figure 9:**
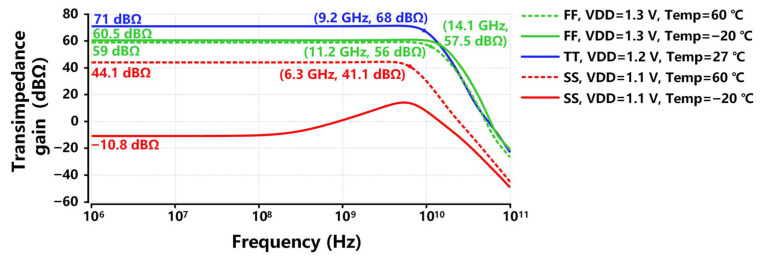
Different cases of post-simulated PVT variations for the transimpedance response of the proposed TIA.

To conduct the best cases and worst cases of PVT variations, Figure 9 exhibits the post-simulated PVT analysis for the transimpedance response of the proposed TIA. The best case of PVT variations occurs at the ff process corner, under the conditions of a 1.3 V supply voltage and a temperature of −20 °C. The proposed TIA achieves a bandwidth of 14.1 GHz and a gain of 60.5 dBΩ. The worst case of PVT variations occurs at the ss process corner, under the conditions of a 1.1 V supply voltage and a temperature of −20 °C, where the TIA exhibits no gain at all. The abnormal transimpedance response at the ss corner is mainly due to increased poly-silicon resistances, which raise the gain of the front-end stages of the proposed TIA, thereby driving the final-stage OB into cut-off operation.

**Figure 10:**
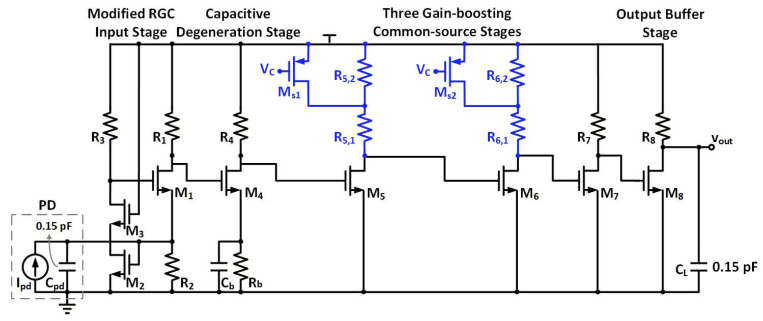
Schematic of the proposed RGC TIA with trimming technique.

As shown in Figure 10, this performance degradation caused by process variations can be optimized via the trimming circuits. In the three gain-boosting CS stages, the original load resistors $R_{5}$ and $R_{6}$ in Figure 2 are actually implemented by $R_{5,1}$ in series with $R_{5,2}$, and $R_{6,1}$ in series with $R_{6,2}$, with their values set to (20a) and (20b), respectively.

(20a) $$R_{5,1}=R_{5,2}=R_{5}/2,$$

(20b) $$R_{6,1}=R_{6,2}=R_{6}/2,$$

Then, $R_{5,2}$ and $R_{6,2}$ are shunted by PMOS switches $M_{s1}$ and $M_{s2}$, respectively. When the circuit is operating well at the ff and tt process corners, Vc is set to the supply voltage, turning off the switches, and the load resistance values remain equal to the original values of $R_{5}$ and $R_{6}$, respectively. When the circuit operates at the ss process corner, Vc is set to ground, turning on the switches and shorting $R_{5,2}$ and $R_{6,2}$, resulting in the load resistance values being halved compared to the original $R_{5}$ and $R_{6}$. This maintains a sufficiently high drain voltage for $M_{5}$ and $M_{6}$, ensuring that all subsequent transistors operate in the saturation region.

**Figure 11:**
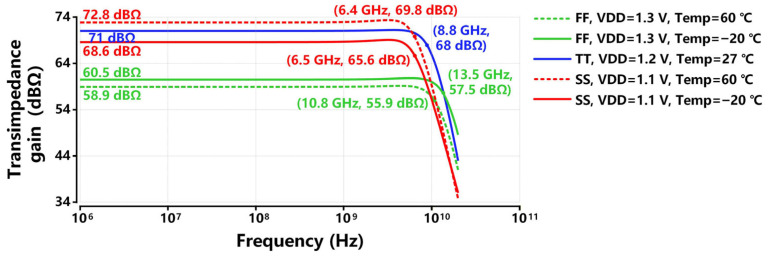
Different cases of post-simulated PVT variations for the transimpedance response of the proposed TIA with trimming technique.

To evaluate the impact of switches on the performance of the proposed TIA circuit, Figure 11 exhibits the post-simulated PVT analysis for the transimpedance response of the proposed TIA with the trimming technique. When simulated at the tt and ff process corners, the PMOS switches are turned off by setting Vc to the supply voltage. The best case of PVT variations occurs at the ff process corner, under the conditions of a 1.3 V supply voltage and a temperature of −20 °C. The proposed TIA achieves a bandwidth of 13.5 GHz and a gain of 60.5 dBΩ. When simulated at the ss process corner, the PMOS switches are turned on by setting Vc to ground. The worst case of PVT variations occurs at the ss process corner, under the conditions of a 1.1 V supply voltage and a temperature of 60 °C. The proposed TIA achieves a bandwidth of 6.4 GHz and a gain of 72.8 dBΩ. Since a bandwidth is about 60~70% of the data rate as recommended for high-speed TIAs [30], the worst-case bandwidth of the proposed TIA can still meet the requirements of 10 Gb/s applications.

**Figure 12:**
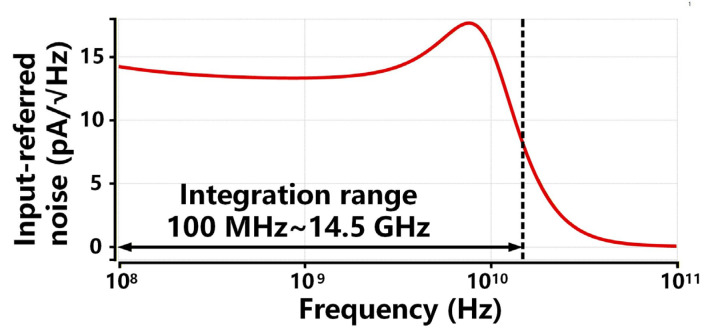
The power spectral density of input-referred noise of proposed TIA design.

Figure 12 presents the input-referred noise current spectral density of the proposed TIA from 100 MHz to 100 GHz. The input-referred noise current can be calculated by dividing the integrated output noise voltage by the TIA’s transimpedance gain, as exhibited by

(21) $$i_{n}^{rms}=\frac{1}{R_{T}}\sqrt{\int_{0}^{{BW}_{noise}}i_{n,out}^{2}(f)df},$$

where ${BW}_{noise}$ represents the equivalent noise bandwidth (ENBW) of the proposed TIA. For typical single-pole low-pass filter systems, ${BW}_{noise}$ is π/2 times the −3 dB bandwidth ${BW}_{-3dB}$. Accordingly, the calculated input-referred noise current $i_{n}^{rms}$ is 1.76 μ A within the integral range from 100 MHz to 14.5 GHz. Therefore, the average input-referred noise current density can be derived as

(22) $$\bar{i_{n,in}}=\frac{i_{n}^{rms}}{\sqrt{{BW}_{-3dB}}},$$

The calculated average input-referred noise is about 18.3 $pA/\sqrt{Hz}$. In the proposed design, the single-stage cascode feedback, capacitive degeneration, and gain-boosting techniques are adopted to enhance the bandwidth and gain while minimizing the noise. Thanks to the noise analysis as derived in Section 2.5, the sizing of the noise-critical devices in the proposed TIA is carefully optimized for noise reduction. The noise performance of the proposed TIA design is about 40~61% better than the existing RGC TIA designs in [23,24,25,26]. Compared with [18], this work achieves comparable noise performance with a higher bandwidth and wider integration range.

**Figure 13:**
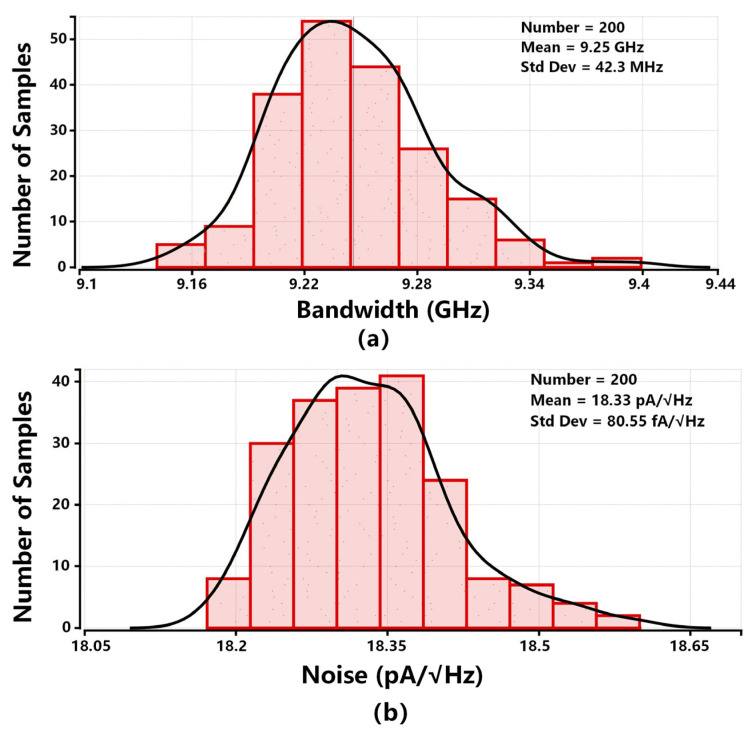
200-iteration Monte-Carlo simulation: (**a**) −3 dB bandwidth, (**b**) noise.

To further verify the impact of process variation, Figure 13 exhibits a 200-iteration Monte-Carlo simulation for −3 dB bandwidth and average input-referred noise. Figure 13a indicates the worst-case value of the bandwidth is 9.16 GHz. The standard deviation of the Monte-Carlo simulation results is 42.3 MHz, which is smaller than 1% of 9.25 GHz, the mean value of the −3 dB bandwidth of the proposed TIA. Figure 13b shows the worst-case value of the input-referred noise is 18.6 $pA/\sqrt{Hz}$. The standard deviation of the Monte-Carlo simulation results is 80.55 $fA/\sqrt{Hz}$, which is smaller than 1% of 18.33 $pA/\sqrt{Hz}$, the mean value of the average input-referred noise of the proposed TIA. Thanks to the careful design of the sizes and parameters of the critical devices in Section 2, a robust broad-bandwidth and noise performance of the proposed TIA subject to the process variation is achieved.

**Figure 14:**
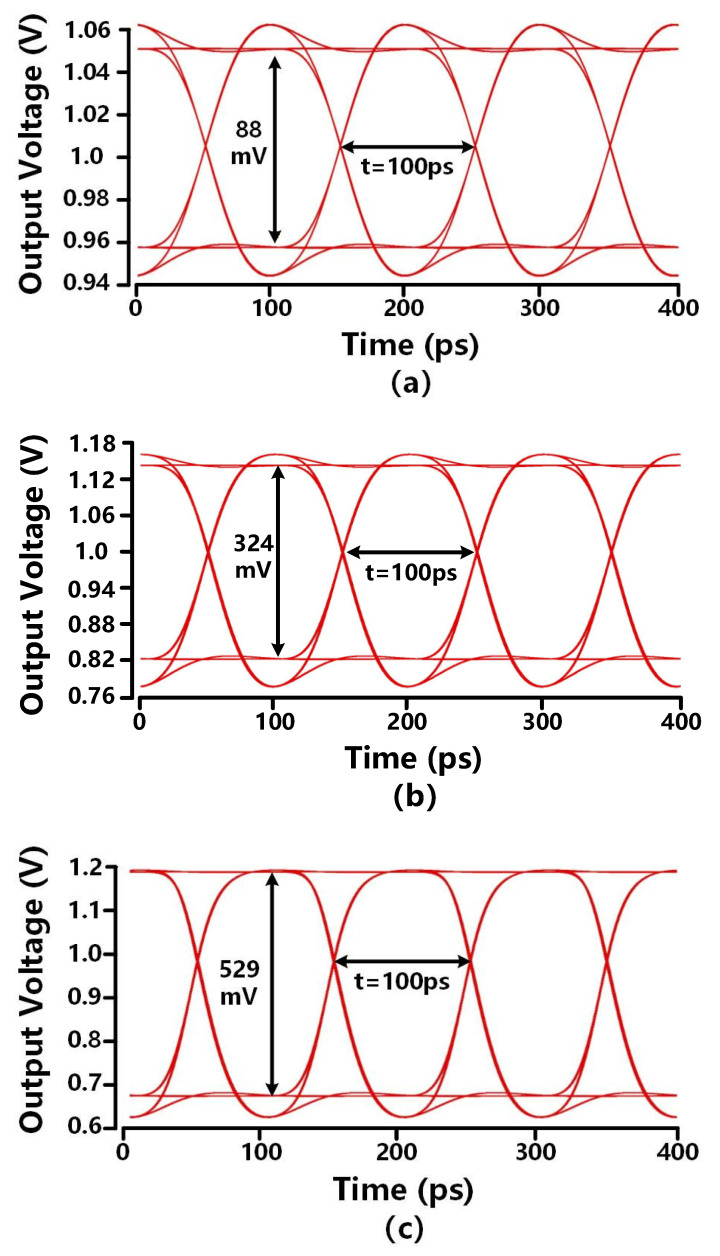
The output eye diagram of proposed TIA design with 10 Gb/s 2^31^-1 PRBS for (**a**) 25 μA (**b**) 100 μA (**c**) 200 μA input current.

Figure 14 depicts the output eye diagram of the proposed TIA with a 10 Gb/s 2^31^-1 pseudorandom binary sequence (PRBS) for different input currents. The 100 ps eye width aligns with the period of the 10 Gb/s signal. The eyes are opened at 88 mV, 324 mV, 529 mV for 10 Gb/s pseudo-random input currents of 25 μA, 100 μA, and 200 μA, respectively. Hence, the proposed TIA provides the input current dynamic range of 18 dB for 10 Gb/s applications. In terms of the dynamic range, its lower limit is determined by noise and signal-to-noise ratio (SNR), while its upper limit is reached when the signal approaches saturation. The calculated input-referred noise current of this work is 1.76 μA. To meet a bit error rate (BER) of 10^−12^, the SNR must be greater than 14, leading to a lower limit of 25 μA peak-to-peak current (Ipp) for the input dynamic range. When Ipp is 200 μA, the upper edge of the eye flattens, which equals the supply voltage, as shown in Figure 14, thus defining the upper limit of the dynamic range. The relatively narrow dynamic range is mainly attributed to the high gain of this work. As this work is a study of pre-TIA, for a specific application requiring higher dynamic range, variable gain technology can be incorporated to make a more complete AFE design to further expand the input dynamic range.

The waveform in Figure 14 shows the proposed TIA circuit exhibits a wide-open, distortion-free eye, which indicates a good signal quality. Since a bandwidth is about 60~70% of the data rate as recommended for high-speed TIAs [30], the bandwidth requirement of 10 Gb/s applications is approximately 7 GHz. The signal quality improvement of this design is primarily attributed to cascode feedback and capacitive degeneration, which reduce $R_{in}$ and further extend bandwidth, respectively. The achieved bandwidth of 9.2 GHz is 1.3 times higher than the requirement for 10 Gb/s applications, providing a sufficient design margin for the 10 Gb/s specification.

**Figure 15:**
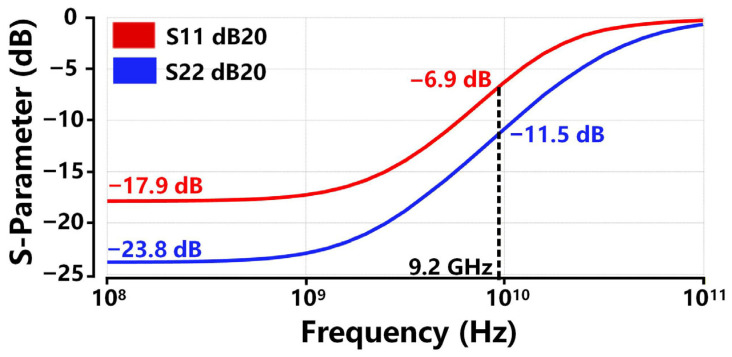
Simulated impedance matching performance of the proposed TIA.

Figure 15 shows the impedance matching performance of the proposed TIA across the frequency range of 100 MHz to 100 GHz, where S11 and S22 denote the reflection coefficients at the TIA’s input and output ports, respectively. The points on the S-parameter curves indicate the simulated S11 and S22 values at 100 MHz are lower than −15 dB and −20 dB, respectively. S11 and S22 remain less than −5 dB and −10 dB over the −3 dB bandwidth of 9.2 GHz. As the input and output resistance is designed to be 50 Ω, the proposed TIA exhibits excellent impedance matching performance, ensuring efficient signal transmission with minimal reflection loss.

**Figure 16:**
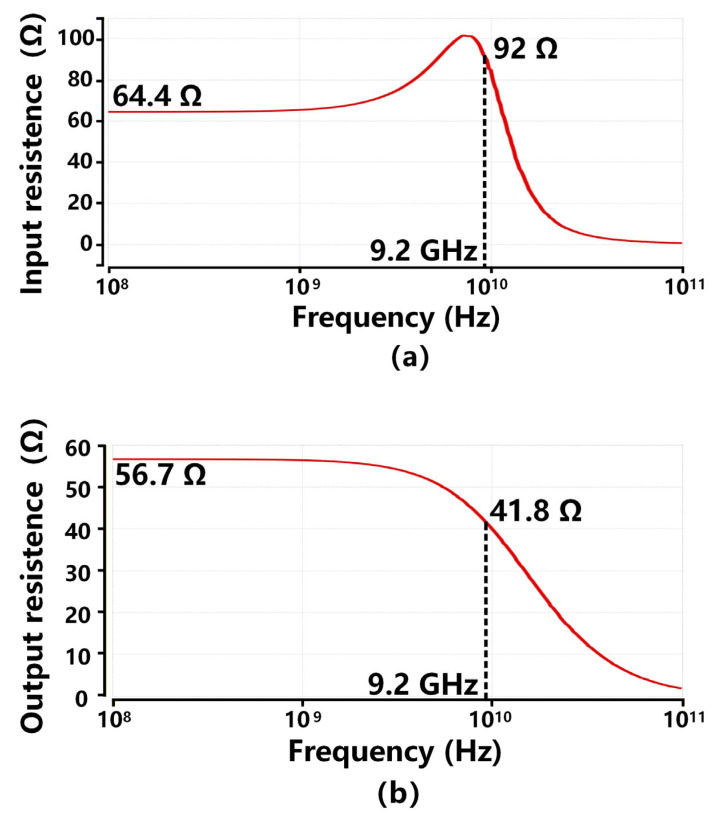
Simulation results: (**a**) the input resistance; (**b**) the output resistance of the proposed TIA.

Figure 16 depicts the simulated input and output resistance from 100 MHz to 100 GHz. Figure 16a shows the input resistance of the proposed TIA is 64.4 Ω at 100 MHz and 92 Ω at 9.2 GHz. This performance achievement benefits from the single-stage cascode feedback in the RGC input stage, which increases the input node pole frequency and reduces the impact of the large $C_{PD}$ at the input node on bandwidth restriction, as compared to the existing inverter-based TIA works. Figure 16b indicates the output resistance of the proposed TIA remains lower than 60 Ω over the −3 dB bandwidth of 9.2 GHz. This performance improvement is mainly due to the output buffer, whose load resistor $R_{8}$ is specifically designed to be 60 Ω for impedance matching. Furthermore, the low output resistance also boosts the second lowest pole frequency, thus extending the overall bandwidth.

Table 3 compares the performance of the proposed TIA design against prior designs in the literature. For a fair comparison, the Figure of Merit (FOM) employed here is the FOM widely used in [16,31] that is normalized by supply voltage (V), as depicted by

(23) $$FOM=\frac{Gain(\Omega )\times BW(GHz)\times C_{PD}(pF)\times Supply(V)}{Power(mW)\times Noise(pA/\sqrt{Hz})},$$

As shown in Table 3, the proposed inductorless design achieves a notably higher FOM of 48.6, reaching 25 dB higher gain, 61% lower noise, and 72% lower power than the state-of-the-art design [26]. This is because the combination techniques of single-stage cascode feedback, capacitive degeneration, and gain boosting relax the stringent trade-off among bandwidth, gain, and noise in advanced CMOS processes. Compared to the inverter-based TIA designs in [16], this work achieves almost the same FOM while consuming 8% less power. Compared with [18], this work achieves comparable noise performance with superior bandwidth, gain, and power. The proposed RGC TIA possesses a 7.4×~243× higher FOM with lower noise and power consumption against the prior RGC TIA designs in [23,24,25,26].

## 4. Conclusions

This paper presents a novel RGC TIA design that achieves high bandwidth, low input-referred noise, and low power consumption in an advanced CMOS process. Through the combination of single-stage cascode feedback, capacitive degeneration, and gain-boosting technologies, the proposed RGC TIA significantly enhances the −3 dB bandwidth while reducing noise and power consumption, which is suitable for emerging optical applications such as Lidar, short-range optical communication, and optical integrated sensing and communication.

## Figures and Tables

**Table 1:** Comparison of bandwidth limiting key factors between the proposed cascode-feedback RGC input stage and the conventional RGC Structure.

	Conventional RGC Structure	Cascode-Feedback RGC Input Stage
Feedback gain	$A\approx -g_{m2}R_{3}/2$	$A^{\prime }\approx -g_{m2}R_{3}=2A$
Input impedance	$R_{in}=\frac{1}{g_{m1}(1+A)}$	$R_{in}^{\prime }=\frac{1}{g_{m1}(1+2A)}$
Miller capacitance	$C_{M}=(1+A)C_{gd2}$	$C_{M}^{\prime }=(1+g_{m2}/g_{m3})C_{gd2}\approx 2C_{gd2}$

**Table 2:** Value of dimensions for the proposed TIA circuit.

Component	Size	Component	Size
$M_{1}$	16μm/40 nm	$R_{1}$	520Ω
$M_{2}$	20μm/40 nm	$R_{2}$	810Ω
$M_{3}$	12μm/40 nm	$R_{3}$	800Ω
$M_{4}$	8μm/40 nm	$R_{4}$	400Ω
$M_{5}$	8μm/40 nm	$R_{5}$	400Ω
$M_{6}$	8μm/40 nm	$R_{6}$	820Ω
$M_{7}$	8μm/40 nm	$R_{7}$	400Ω
$M_{8}$	16μm/40 nm	$R_{8}$	60Ω
$C_{b}$	100 fF	$R_{b}$	190Ω

**Table 3:** Performance comparison of 10 GB/s TIAs.

Design	ISCAS [16] ^b^	Electronics [24] ^b^	TCAS-I [18] ^a^	TCAS-I [23] ^a^	Electronics [26] ^b^	MEJ [25] ^b^	This Work ^b^
Technology	40 nm	0.18 μm	0.18 μm	0.13 μm	65 nm	0.13 μm	40 nm
Topology	Inverter	RGC	RGC	RGC	RGC	RGC	RGC
Supply (V)	1.1	1.8	1.8	1.5	1	1.3	1.2
BW (GHz)	5.2 ^c^	10 ^c^	8 ^c^	7	11.4 ^c^	10.1	9.2
PD cap. (pF)	0.075	0.15	0.25	0.25	0.1	1	0.15
Gain (dBΩ)	75	41	53	50.1	46	47	71
Noise ($pA/\sqrt{Hz}$)	6.9	30.7	18	31.3	46.6	42	18.3
Power (mW)	7.15	10.7	13.5	7.5	23.9	12.2	6.6
FOM	48.9	0.92	6.6	3.6	0.2	5.7	48.6

^a^ Measurement result. ^b^ Post-layout simulation result. ^c^ Using inductive peaking.

## Data Availability

The data that support the findings of this study are available from the corresponding author upon reasonable request.
